# Data-Driven Derivation of Molecular Substructures That Enhance Drug Activity in Gram-Negative Bacteria

**DOI:** 10.1021/acs.jmedchem.1c01984

**Published:** 2022-04-15

**Authors:** Dominik Gurvic, Andrew G. Leach, Ulrich Zachariae

**Affiliations:** † Computational Biology, School of Life Sciences, University of Dundee, Dow Street, Dundee DD1 5EH, United Kingdom; ‡ Division of Pharmacy and Optometry, University of Manchester, Oxford Road, Manchester M13 9PL, United Kingdom; § Medchemica Limited, Mereside, Alderley Park, Macclesfield, SK10 4TG, United Kingdom

## Abstract

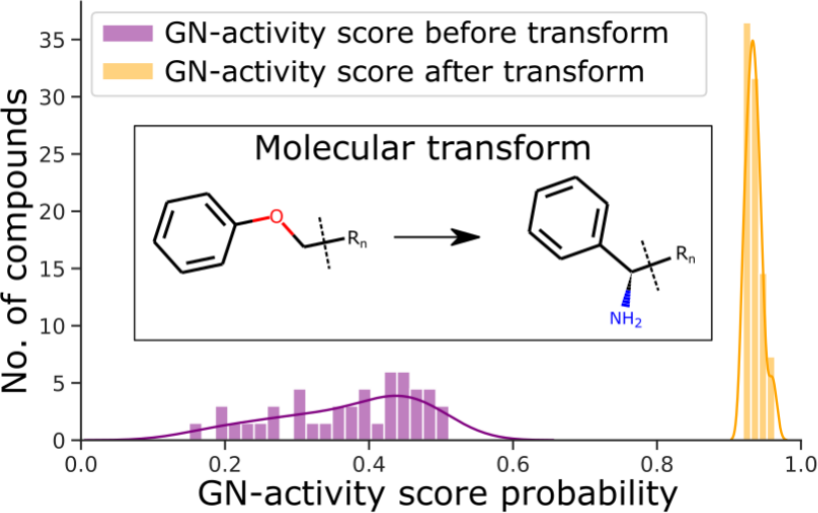

The complex cell envelope of Gram-negative bacteria creates a formidable barrier to antibiotic influx. Reduced drug uptake impedes drug development and contributes to a wide range of drug-resistant bacterial infections, including those caused by extremely resistant species prioritized by the World Health Organization. To develop new and efficient treatments, a better understanding of the molecular features governing Gram-negative permeability is essential. Here, we present a data-driven approach, using matched molecular pair analysis and machine learning on minimal inhibitory concentration data from Gram-positive and Gram-negative bacteria to uncover chemical features that influence Gram-negative bioactivity. We find recurring chemical moieties, of a wider range than previously known, that consistently improve activity and suggest that this insight can be used to optimize compounds for increased Gram-negative uptake. Our findings may help to expand the chemical space of broad-spectrum antibiotics and aid the search for new antibiotic compound classes.

## Introduction

The majority of severely drug-resistant bacterial infections are caused by Gram-negative (GN) bacteria, which account for two-thirds of the priority list of highly drug-resistant pathogens published by the World Health Organization (WHO) in 2017. Critical priority GN bacteria include *Acinetobacter baumannii*, *Pseudomonas aeruginosa*, *Enterobacteriaceae*, and *Klebsiella pneumoniae*.^1^ In 2019 and 2020, the WHO concluded that the antibiotics currently in the development pipeline will not be sufficient to combat the large spectrum of drug-resistant pathogenic bacteria. Since 2017, 11 new antibiotics have been approved for use, nine of which are closely related derivatives of existing antibiotic classes and hence are subject to cross-resistance.^2,3^ There is thus an urgent medical need to develop additional broad-spectrum antibiotics, particularly of new classes, which are capable of evading existing resistance pathways.

In the case of GN bacteria, bioactivity is impeded by a high level of intrinsic resistance, arising from the poor drug permeability of the GN cell envelope. Especially the outer membrane, a unique feature of GN bacteria, presents a major barrier to drug uptake. Antibiotics can traverse the outer membrane either via porins, trimeric pore proteins that usually generate passageways for hydrophilic molecules, or, in some cases, directly via lipid-mediated membrane diffusion.^4−7^ The physicochemical character and pore dimensions of porin channels are widely thought to restrict the maximum drug influx rates achievable in GN bacteria, and mutations in the channels, as a result of acquired resistance, can further reduce uptake rates.^8,9^ Tripartite efflux pumps function synergistically with the low inward permeability by actively expelling a broad range of drugs from the periplasm, a buffer volume between the outer and inner membranes in GN bacteria. Overcoming the permeability barrier in the cell envelope of GN pathogens has been widely recognized as the key obstacle to the development of new broad-spectrum antibiotics, active against both Gram-positive (GP) and GN bacteria. However, the physical and chemical underpinnings of drug permeation into GN bacteria are poorly understood.^10−14^

Over the past two decades, efforts have been made to derive general physicochemical composition rules to guide the design of new antibiotics by analyzing existing drugs. Most of the early studies in this area were based on small datasets and often focused on antibiotics available on the market at the time.^14−16^ Through the development of liquid chromatography–tandem mass spectrometry (LC-MS/MS) techniques, which allow accurate bacterial accumulation assays, research into GN permeation rules gained further momentum.^12,17,18^ Although multiple studies which included analyses of both external and proprietary datasets have since provided insights into GN activity, there appears to be no consensus regarding the key physical and chemical determinants of compound uptake.^19^ In a recent landmark study, it was shown that the addition of terminal amine groups, among other factors, considerably improved the GN permeability of GP-active antibacterial compounds.^18,20^ However, adding terminal amine groups to a given lead compound will not always be feasible, and it is therefore essential to broaden the understanding of chemical space accessible to enhance GN permeation. This would not only help to accelerate anti-bacterial drug design but also constitute an important step forward toward tackling the widely encountered problem of efficient drug permeation into cells more generally.

In recent years, the field of cheminformatics has experienced a significant boost from the adoption of machine learning (ML) approaches in areas including lead generation, lead optimization, and physicochemical property prediction.^21^ ML modeling has previously been applied to antibiotic design, using public as well as proprietary datasets, potentially opening new avenues toward developing a new generation of antibiotics.^18,21−24^ Here, we build on these recent milestones to shed light on the major chemical determinants of GN drug activity and formulate chemical rules to enhance activity, focusing in particular on permeability across the GN cell envelope. We used a dataset composed of 1887 compounds with associated minimal inhibitory concentration (MIC) data in the GN bacterium *Escherichia coli* and the GP bacterium *Staphylococcus aureus* after a strict curation process to yield a proxy for permeation, followed by a combination of ML-based data-driven activity prediction, matched molecular pair analysis (MMPA), and independent validations on experimental data. While confirming the usefulness of terminal amine groups to enhance GN permeability, our results reveal a broader variety of chemical modifications that increase GN activity, and we delineate an approach to optimize compound property prediction from a limited-size, but rigorously curated, publicly available dataset.

## Results and Discussion

### Curation and Matched Molecular Pair Analysis of MIC Data

We retrieved single-point MIC data for 19 417 compounds from the CDD and a further 9645 MIC datapoints from CO-ADD. The data were curated to form a proxy for GN permeation according to the criteria summarized in Table 1. Applying these criteria led to 934 compounds labeled as “1”, GN-active class, and 953 labeled as “0”, GN-inactive class (GN-activity dataset 1; Table 2).

**Table 1:** Curating Compounds Based on Activity against *S. aureus* vs *E. coli*

pathogen	compound 1	compound 2		compound 1887
*S. aureus*	pMIC ≥ 5	pMIC ≥ 5	...	pMIC ≥ 5
*E. coli*	pMIC ≥ 5	pMIC < 5		pMIC ≥ 5
*final label*^a^	*1*	*0*		*1*

a Compounds that are active against both pathogens above a threshold MIC are labeled “1” (GN-permeable), and compounds that are active against *S. aureus* but inactive against *E. coli* are labeled “0” (GN-impermeable).

**Table 2:** Number of *S. aureus* and *E. coli* MIC Datapoints Initially Retrieved from the CO-ADD and CDD Databases and Number of GN-Inactive and GN-Active Compounds Representing Permeation after Curation of the Initial Data

**Initial MIC Dataset**			
*Staphylococcus aureus*		*Escherichia coli*	
data origin	no. of data points	data origin	no. of data points
CDD	11 428	CDD	7 989
CO-ADD	4 934	CO-ADD	4 711
*total*	*16 362*		*12 700*


**Curated MIC Dataset (GN-Activity Dataset)**			
GN-inactive	953	GN-active	934

**Table 3:** Overview of the Datasets Used for MMPA in This Study: Number of Derived Matched Pairs and Number of Significant Molecular Transformations Remaining after Performing Paired *t* tests and Secondary Filtering from Each Dataset

dataset	total compounds	total pairs	unique transforms	significant transforms
GN-activity-1	1887	4922	4162	0
ENM_1	2.1M	240k	164k	499
ENM_2	460k	360k	271k	1057
ENM_3	43k	1M	735k	1149
*total*	*2.6M*	*1.6M*	*1.1M*	*2705*

**Figure 1:**
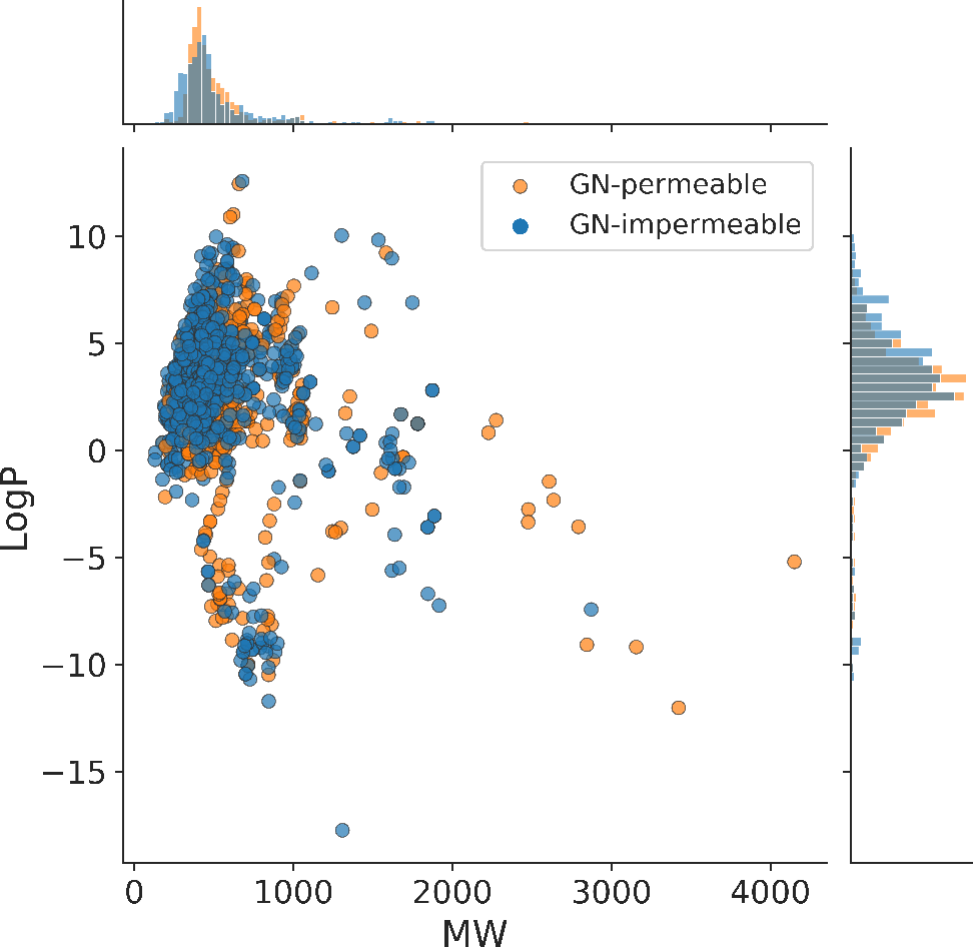
Chemical space occupied by GN-active (permeable, label “1”) and GN-inactive (impermeable, label “0”) compounds within GN-activity dataset 1 according to our curation, represented by their molecular weight (MW) and hydrophobicity (log *P*).

We first examined the coverage of property space for the two distinct classes of molecules. Figure 1 shows the distribution of molecular weight (MW) and hydrophobicity (log *P*) for GN-active vs inactive compounds. The GN-active molecules display a shift toward a smaller average log *P* value ( ≃ 2.41,  ≃ 2.96), with the two distributions exhibiting a significant (*p*-value = 1 × 10^–4^) difference from each other, although on the graph, the hydrophobicity regions occupied by the two types of molecules are largely inseparable.

Furthermore, GN-active compounds exhibit a slight tendency toward larger average MW, ( ≃ 530.56,  ≃ 519.24), although no significant difference between the MW distributions is observed here (*p*-value = 0.433).

Previous work, based on the analysis of less abundant compound datasets, has suggested that GN-permeable molecules tend to be more hydrophilic and smaller than GN-impermeable molecules. This effect has mainly been attributed to the selection criteria for permeating porin channels in the GN outer membrane.^14,19^ Porins possess a highly hydrophilic inner pore lining and a narrow, charged eyelet region which imposes an additional size, or MW, limitation on the spectrum of translocated molecules.^6,25^ According to our present analysis of 1887 compounds, however, neither MW nor log *P* can serve as primary separator or predictor of activity or permeability across the GN cell envelope, despite the small shift observed in log *P*. Recently, interactions between different classes of antibiotics and lipopolysaccharides in the GN bacterial outer membrane have been characterized, highlighting direct pathways into the outer membrane that do not involve porins.^26^ Similarly, it has been shown that permeating antimicrobials bypass the porins in the GN bacterial pathogen *P. aeruginosa*.^7^ A greater diversity of inward permeation pathways than previously thought could, accordingly, explain the absence of clear MW or log *P* constraints on GN activity in our analysis and be responsible for the lack of consensus among previous studies.^19^ We next performed an initial cycle of MMPA on GN-activity dataset 1 to identify molecular transformations that are associated with a change in MIC. To confine our further analyses exclusively to transformations with statistically significant effects on MIC, we used a paired *t* test on MIC distributions that differ by the same transform and set a *p*-value threshold of ≤0.05. The transforms were further filtered for a positive *t*-statistic, leaving only transforms that lead to an increase in activity, i.e., smaller MIC or larger pMIC values. We applied a Benjamini–Hochberg correction to all sets of *t* tests carried out in this study to control for the expected false discovery rate.^27^

Table 3 shows that the numbers of total pairs and unique transforms are of similar magnitude within GN-activity dataset 1, indicating that there is only a small number of unique transforms with multiple repeats. Further analysis, based on the previously defined conditions, showed that none of the transforms in GN-activity dataset 1 passed the strict significance threshold we set. We therefore next aimed to expand the initial compound dataset by generating synthetic GN-activity data, focusing on permeation by the previous curation step, through ML modeling.

### Generating Synthetic Data

Initial hyperparameter optimization on a training dataset (85% of compounds in GN-activity dataset 1, *n* = 1604; 807 GN-active, 797 GN-inactive molecules), with a test set retained separately, resulted in the following parameters for all models used in the present study: hidden size of the neural network layers, 1700; number of message passing iterations, 6; dropout probability, 0.05; and number of feed-forward layers, 1.^44^ Using these parameters, a classifier consisting of an ensemble of five *Chemprop* models was trained and 5-fold cross-validated, leading to a resulting overall training score of AUC = 0.92 ± 0.01. A recommended built-in method for additional normalized 2D rdkit features was used in parameter optimization and during training.^45^ Finally, the ensemble was tested on the remaining 15% of compounds (*n* = 283; 127 GN-active, 156 GN-inactive), achieving a test score of AUC = 0.98. The trained model was then used to carry out predictions of GN-activity scores on a scale between 0 and 1 for each compound within three external datasets, ENM_1–ENM_3.

### Matched Molecular Pair Analysis of Synthetic Data

Each of the datasets, ENM_1, ENM_2, and ENM_3, containing compounds with predicted GN-activity scores, was analyzed separately using MMPA. The molecular transformations found through MMPA were then combined into a single dataset and subjected to statistical testing (paired *t* tests), which yielded 2705 significant transforms. The average difference in activity is 0.19, while the average number of repeats we observe for each transformation is 7.75.

**Figure 2:**
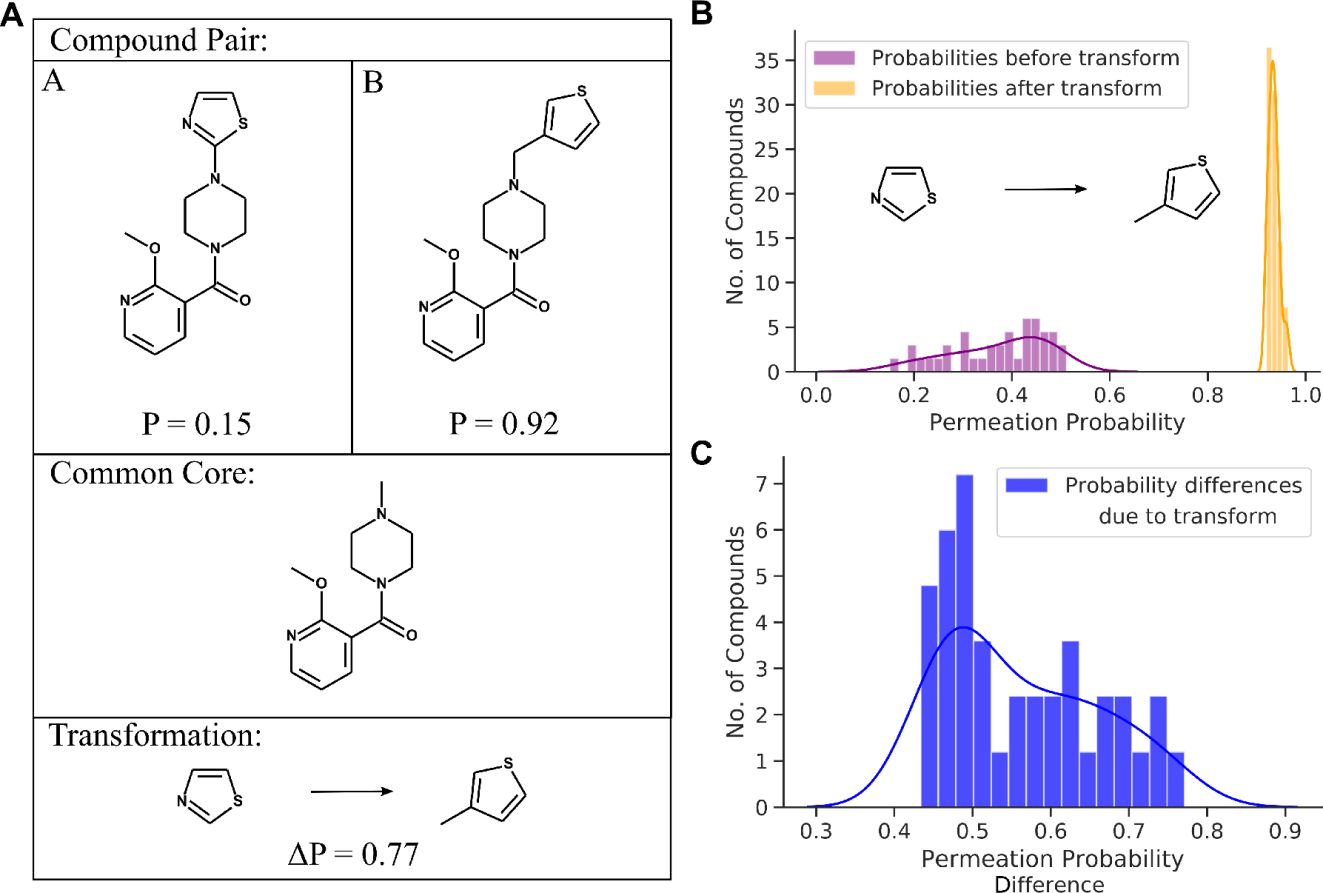
(A) Matched molecular pair analysis (MMPA) consists of finding pairs of molecules (“A” and “B” in the diagram) that only differ by a small structural change, referred to as chemical “transformation”, while maintaining a common core. Since every molecule is assigned an individual GN-activity (or permeability) score *P*, the transformation can be associated with the difference in the score, Δ*P*. (B) Distribution of all predicted GN-activity scores among molecules containing the depicted transformation in association with a maintained core (“A”, low-permeability partner in purple, and “B”, high-permeability partner in orange). (C) Distribution of the score difference between all matching pairs with the same transformation.

We next analyzed the chemical nature of the transforms, aiming to detect molecular substructures that consistently enhance or decrease the predicted GN-activity score throughout the whole dataset. For each transform, we identified functional groups and moieties present in the left-hand side (LHS) and right-hand side (RHS) of each transform separately (cf. compound pair shown in Figure 2, left and right of the transformation arrow). The LHS collection of substructures describe the lower GN-activity member of each transform and the RHS substructures characterize its higher GN-activity counterpart. The substructures were then compared to a predefined list consisting of 153 descriptors of functional groups and moieties in SMARTS notation (Table S1A).^28^

We then restricted the number of substructural descriptors to those undergoing a significant change in the transformations by carrying out *t* tests (*p*-value ≤ 0.01) on distributions prior to and after the transformation, followed by Benjamini–Hochberg correction.^27^ Subsequently, we focused on transformations resulting in an increase in GN activity, but in principle, due to the nature of MMPA, every transform examined can be reversed to represent statistically significant reductions in predicted activity. Descriptors representing generic substructures (e.g., arene, heteroarene, alkyne, alkene, benzene ring, and aza-arene) were discarded since they describe largely unspecific changes to the molecules encountered regularly in most transformations, which mostly do not contain useful information on feasible modifications. Taken together, these steps yielded 15 key descriptors that were retained for further analysis (Table S1B).

### Molecular Transformations Associated with Increased GN Activity

We analyzed every substructural descriptor that was linked to a significant enhancement of the predicted GN activity across the set of molecular transformations, as shown in Table 4. We then determined the average increase within each subset (), whether addition or removal of the respective chemical moiety led to this increase (+ /−), the number of transformations in which a particular moiety change was observed (*repeats*), and the most commonly exchanged counter-moieties in the respective transformations (*opposite moieties*, see below).

**Table 4:** MMPA-Generated Key Molecular Transformations Shown to Significantly Affect GN-Activity Scores of Synthetically Generated Data^a^

entry	main moiety	+/–	± std	repeats	opposite moiety 1	opposite moiety 2	opposite moiety 3
1	primary amine	addition	0.42 ± 0.25	61	ether [25%]	carbonyl [20%]	secondary amine [10%]
2	lactone	removal	0.41 ± 0.08	35	secondary amine [14%]	tertiary amine [9%]	ether [6%]
3	ester (carboxylate ester)	removal	0.39 ± 0.16	56	secondary amine [9%]	(ether, primary amine) [7%]	tertiary amine [7%]
4	carbonyl	removal	0.37 ± 0.17	96	thiophene [17%]	primary amine [13%]	aryl chloride [9%]
5	nitrile	removal	0.33 ± 0.13	255	aryl chloride [22%]	(thiophene, ether) [12%]	aryl fluoride [5%]
6	thiophene	addition	0.32 ± 0.15	304	(nitrile, secondary amine) [9%]	aniline [8%]	carbonyl [5%]
7	tertiary carboxamide	removal	0.31 ± 0.17	31	thiophene [42%]	aryl chloride [23%]	secondary carboxamide [19%]
8	aryl chloride	addition	0.28 ± 0.16	279	nitrile [20%]	tertiary amine [6%]	aryl fluoride [4%]
9	secondary amine	addition	0.24 ± 0.13	306	tertiary amine [16%]	ether [3%]	nitrile [3%]
10	tertiary amine	removal	0.22 ± 0.09	292	secondary amine [17%]	(aryl chloride, aryl fluoride) [6%]	alkanol [7%]
11	α,β-unsaturated carbonyl	removal	0.21 ± 0.14	33	thiophene [18%]	secondary amine [15%]	aryl chloride [12%]
12	aryl fluoride	addition	0.20 ± 0.13	154	nitrile [16%]	tertiary amine [12%]	alkanol [3%]

a Listed are the molecular replacements that correlate with a strong increase of the predicted GN activity, most likely due to improved permeability according to our initial curation, by either addition or removal of each specific main moiety (+/−). The top three exchange counterparts in the matched molecular pairs are shown as opposite moieties.  denotes the average change of the GN-activity score (limits from 0 to 1), linked to the respective transformations with associated standard deviation. “Repeats” indicates the number of transformations that contain the “main moiety”.

To illustrate our approach, the exemplar moiety, thiophene (Table 4, entry 7), was encountered in 304 out of the 2705 transforms (“repeats”). Taken together, these chemical transformations led to an average increase of ∼0.32 in the activity score. Here, this marked increase was observed for the *addition* of a thiophene moiety (+/−). In the transforms, thiophene most often replaced nitrile or secondary amine groups (joint opposite moiety 1, in 9% of the cases each), aniline (opposite moiety 2, in 8% of cases), and carbonyl groups (opposite moiety 3, in 5% of all cases).

Table 4 shows that, on average, the addition of a primary amine group exerts the largest effect on increasing the activity score ( ≃ 0.42, represented by 61 transforms). The effect of primary amines is larger than that of secondary amines ( ≃ 0.24), represented in 306 transforms. These results are in excellent agreement with the previous findings of Richter et al., who reported that primary amine groups increased GN permeability after examining a set of 180 chemically diverse compounds tested on *E. coli*.^18^ This underscores the validity of our approach based both on experimentally recorded and synthetic data, and using curated bioactivity data as a proxy for cell wall permeability.

In our analysis, however, a range of further moieties is shown to be associated with a substantial change in activity, similar to the level seen for primary amines (Table 4). For example, the removal of ester groups (56 repeats) and their cyclic subset, lactones (35 repeats), markedly improves the activity score (by  ≃ 0.41 and ≃0.39, respectively). While large, 14–16-membered lactone rings are known structural elements of the natural antimicrobial class, macrolides, the lactones examined in our transformations are mostly smaller rings with up to 6 members. The substitution of carbonyl ( ≃ 0.37), nitrile ( ≃ 0.33), and carboxamide groups ( ≃ 0.32) improves GN activity by a similar extent. Notably, the addition of thiophene groups is only slightly less effective in increasing activity than the addition of a primary amine group ( ≃ 0.32). Furthermore, adding an aryl chloride group yields an activity increase of comparable magnitude ( ≃ 0.28), while the addition of secondary amines or aryl fluorides is also linked to a marked enhancement of GN activity ( ≃ 0.24 and  ≃ 0.20, respectively; Table 4). While tertiary amines appear to be a second replacement choice for two moieties that are negatively correlated with improved GN activity, on average they are themselves negatively correlated, especially when compared to primary and secondary amines, which suggests that replacing tertiary amines with secondary or primary amines increases the probability of permeation.

Overall, a clear pattern emerges of groups such as primary and secondary amines, and thiophenes and aryl halides, which have large positive effects on GN activity, especially when they replace substituent groups containing carbonyl oxygen (including esters, lactones, and carboxamides). Our work thus demonstrates that, beyond the addition of primary amines and other nitrogen-containing groups, a range of alternative modifications to a given core molecule are likely to have similarly large effects on GN permeability.

### Independent Test of the Chemical Substitution Rules on Experimental Data

Although our predictive model was initially trained on rigorously curated measured MIC data, the statistical power of the MMPA we performed relies on the use of additional synthetic data. It is important, therefore, to independently validate our results on datasets obtained exclusively from experimentally investigated compounds. We thus screened *in vitro* data from the ChEMBL database for the presence of the patterns we predict to be linked to GN activity and permeation.^29^

The ChEMBL database merges MIC measurements obtained using a range of different assay types and from many different bacterial strains, including those in which bacterial permeation factors such as porins or drug efflux pumps were altered or deleted. The mixed composition of the ChEMBL dataset means that it is not as suited for use as a training set as the more highly curated databases, CDD and CO-ADD; however, after a careful manual curation step, the data is arguably appropriate to serve as a test set. We therefore collected all available inhibition data for both *S. aureus* and *E. coli* from ChEMBL, standardized all deposited inhibition units into pMIC, removed duplicated datapoints, and deleted datapoints resulting from assays involving mutated strains or strains with induced antibiotic susceptibility. This resulted in 24 102 datapoints for *E. coli* and 35 802 for *S. aureus* (ChEMBL dataset 1). Subsequently, this pMIC data was further curated to serve again as proxy for GN permeation data according to our previously used approach (see Methods, Data Curation), yielding 5009 GN-active compounds (*E. coli* pMIC ≥ 5; *S. aureus* pMIC ≥ 5) and 2955 GN-inactive compounds (*E. coli* pMIC < 5; *S. aureus* pMIC ≥ 5) (ChEMBL dataset 2).

To ascertain if the chemical transformations identified earlier increase the GN pMIC of a core molecule, we performed MMPA directly on the *E. coli* pMIC values (ChEMBL dataset 1). Separately, MMPA was carried out on the new permeation-proxy data to investigate if the transformations introduce additional GN activity into GP-active molecules (ChEMBL dataset 2). The MMPA was followed by substructure search, matching any functional groups and moieties in the LHS and RHS of each transformation in both datasets to the previously identified activity-enhancing transforms that are likely due to improved permeability.

Table 5 displays the statistics we obtained from screening the ChEMBL datasets for each of the main moieties and their exchange counterparts. As shown in the table, the ChEMBL *in vitro* data contains a large number of examples for the molecular substitutions that our previous computational analysis suggested to enhance GN activity and permeation. Intriguingly, 89% (31/35) of the computationally identified transforms indeed increase the *E. coliin vitro* pMIC in ChEMBL dataset 1. Furthermore, 86% (30/35) of the transformations turn at least one compound in the sets from GN-inactive to GN-active, and in 71% (25/35) of transforms in ChEMBL dataset 2, we find at least one example where the transform modifies a GP-only active compound into a compound that is active against both GP and GN bacteria.

**Table 5:** Validation of Moiety Exchanges Predicted to Improve GN Activity by Screening *In VitroE. coli* MIC Data and Permeation-Proxy Data Curated from ChEMBL for Every Added or Removed “Main Moiety” and Its “Opposite Moiety” Counterpart^a^

main moiety	+/–	exchange moiety	ΔpMIC ± std	pMIC Repeats	inactive → active	GP → GN (repeats)
primary amine	addition	ether	0.83 ± 0.61	225	50	28 (179)
primary amine	addition	carbonyl	1.07 ± 0.71	655	283	122 (396)
primary amine	addition	secondary amine	0.49 ± 0.63	2267	60	32 (2049)
lactone	removal	secondary amine	1.40 ± 0.15	5	0	0 (3)
lactone	removal	tertiary amine	0.78 ± 0.32	71	0	0 (39)
ester (carboxylate ester)	removal	secondary amine	0.81 ± 0.45	21	2	0 (13)
ester (carboxylate ester)	removal	carboxamide	1.23 ± 0.51	28	27	25 (26)
ester (carboxylate ester)	removal	ether	0.55 ± 0.62	26	3	1 (13)
ester (carboxylate ester)	removal	primary amine	0.89 ± 0.45	36	12	1 (25)
ester (carboxylate ester)	removal	tertiary amine	0.74 ± 0.35	106	5	4 (51)
carbonyl	removal	aryl chloride	0.67 ± 0.68	48	16	2 (16)
nitrile	removal	ether	0.33 ± 0.55	77	6	5 (34)
carboxamide	removal	thiophene	0.27 ± 0.27	8	0	0 (1)
carboxamide	removal	carboxylic acid	0.59 ± 0.33	42	25	5 (11)
carboxamide	removal	aryl chloride	0.42 ± 0.61	5	1	1 (2)
thiophene	addition	nitrile	–0.04 ± 0.89	9	3	3 (4)
thiophene	addition	secondary amine	–0.23 ± 1.09	31	4	1 (8)
thiophene	addition	aniline	0.42 ± 0.69	16	5	3 (6)
thiophene	addition	carbonyl	0.74 ± 0.40	39	4	2 (15)
tertiary carboxamide	removal	thiophene	0.37 ± 0.26	5	0	0 (1)
tertiary carboxamide	removal	secondary carboxamide	0.5 ± 0.35	35	6	1 (13)
aryl chloride	addition	nitrile	0.16 ± 0.48	53	8	2 (9)
aryl chloride	addition	tertiary amine	0.39 ± 0.65	52	16	2 (4)
aryl chloride	addition	aryl fluoride	0.02 ± 0.50	291	17	5 (49)
secondary amine	addition	tertiary amine	0.43 ± 0.68	1066	142	85 (755)
secondary amine	addition	ether	0.70 ± 0.51	203	53	37 (150)
secondary amine	addition	nitrile	1.19 ± 1.03	62	26	14 (35)
tertiary amine	removal	aryl chloride	0.39 ± 0.65	52	16	2 (4)
tertiary amine	removal	alkanol	0.45 ± 0.51	236	31	18 (99)
α,β-unsaturated carbonyl	removal	thiophene	1.04 ± 0.21	11	0	0 (6)
α,β-unsaturated carbonyl	removal	secondary amine	1.24 ± 0.69	25	4	0 (2)
α,β-unsaturated carbonyl	removal	aryl chloride	1.20 ± 0.74	7	3	0 (2)
aryl fluoride	addition	nitrile	0.17 ± 0.60	64	1	0 (16)
aryl fluoride	addition	tertiary amine	0.79 ± 0.64	56	21	2 (17)
aryl fluoride	addition	alkanol	0.33 ± 0.69	81	14	2 (23)

a As defined in Table 4, identical matched pairs were identified in the ChEMBL datasets. “pMIC repeats” denotes the number of identified pairs, “ΔpMIC” the average change in experimental pMIC, and “inactive → active”, the number of times the change in pMIC changed the core molecule from GN-inactive (pMIC < 5) to GN-active (pMIC > 5) in ChEMBL dataset 1. “GP → GN” displays the number of times a core molecule is altered from GN-inactive to GN-active in the subset, ChEMBL dataset 2, with the number of repeats for the respective pairs in this dataset shown in parentheses. The full distributions of the change in pMIC for every transform are shown in the Supporting Information, Figure S3.

The top row of Table 5 shows 225 occurrences (“pMIC repeats”) in ChEMBL dataset 1 where a primary amine is gained (“main moiety”) in favor of an ether group (“exchange moiety”). These molecular transformations show an average positive increase in the *E. coli* pMIC of 0.83 (ΔpMIC). Furthermore, within those 225 examples, we find 50 cases in which this functional group substitution turns a GN-inactive compound into a GN-active molecule (according to our previously used definition, from activity below 5 pMIC to greater than 5 pMIC; “inactive → active”). In the ChEMBL permeation-proxy dataset (ChEMBL dataset 2), we find 179 examples for the same molecular substitution. In 28 of these cases, a GP-only active compound, by addition of primary amine in favor of an ether, is modified to become a broad-spectrum active compound against both GP and GN bacteria (“GP → GN”).

**Figure 3:**
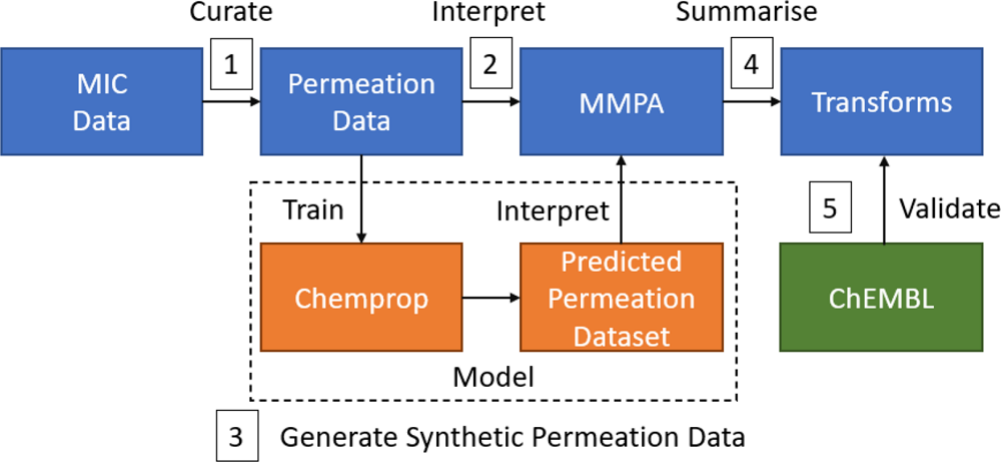
Flowchart of the approach taken to derive properties of GN activity and permeation. (1) Compounds with known MIC values from CDD and CO-ADD were rigorously curated to represent a proxy for GN permeation, yielding GN-activity dataset 1. (2) The GN activity or permeation data was interpreted using MMPA to correlate differences in activity to structural features. (3) To supplement the experimentally recorded data (limited by the size of the input data and the rigorous curation process), a machine learning model was used to predict the GN activity of compounds in large new datasets of 2.6M compounds combined. For these molecules, the predicted GN-activity score replaced recorded MIC/permeability data for MMPA. (4) The results from MMPA were analyzed and grouped into molecular transformations that substantially impact GN activity. (5) The derived transformations were validated by analyzing their effect on experimental GN- and GP-activity datasets retrieved from the ChEMBL database.

**Figure 4:**
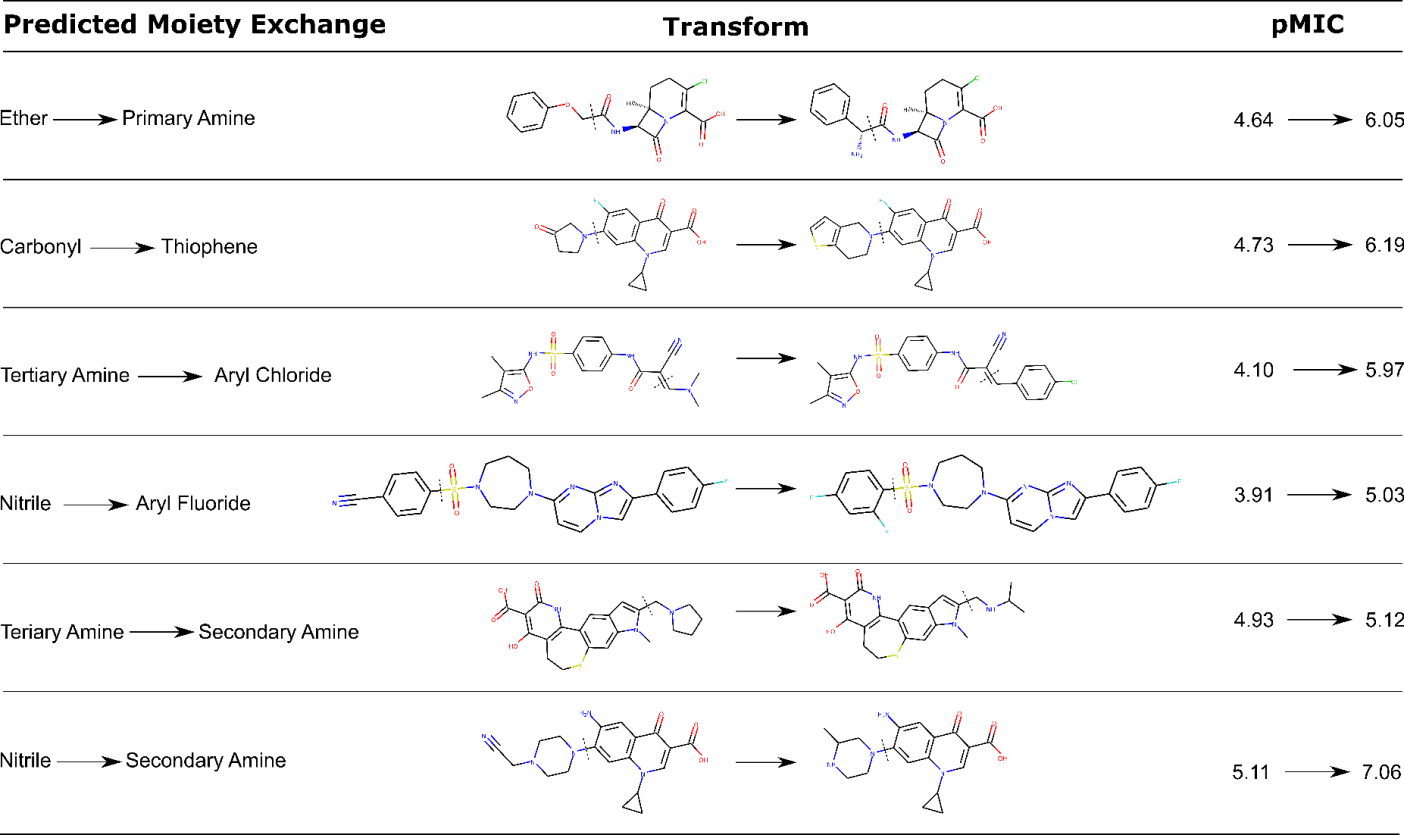
Six example transforms from the ChEMBL datasets where a detected significant fraction exchange leads to increased GN activity. The dotted line indicates where the moiety alteration takes place, while the main molecular core remains identical. The first five examples turn a GN-inactive molecule into a GN-active compound according to our definition, while in the last example, a moderately GN-active molecule is modified into a highly GN-active one. (Note that in some of the cases the transforms contain more than only a change in the functional groups or moieties by which we identified them, but our identified substructures are the common denominator, emerging from large and diverse datasets.)

This independent screen of a large amount of experimental data provides further evidence that the molecular modifications suggested by our computational MMPA enhance GN activity, and likely permeation *in vitro*. The vast majority of our transforms are found to have a substantially positive effect on the *E. coli* pMIC. Only two cases, exchanging thiophene for nitrile or secondary amine, on average resulted in a negative pMIC change. A further five transforms failed to convert inactive compounds into active ones according to our pMIC definition (e.g., removal of lactone in favor of secondary amine). A further nine transforms did not convert compounds from Gram-positive active into Gram-negative active (e.g., removal of tertiary carboxamide in favor of thiophene), represented in the last two columns in Table 5. In two cases, no examples for our predicted exchanges were retrieved from the ChEMBL datasets (ether to lactone and aryl chloride to tertiary carboxamide). Figure 4 displays five examples of compound pairs retrieved from the ChEMBL database in which the identified transforms improve GN activity. In all five examples shown, GN-inactive compounds (pMIC < 5) are rendered GN-active (pMIC > 5) by the moiety exchange. In addition, we show an exemplar transform, in which the molecular exchange turns a compound with borderline GN activity into a molecule with high GN activity.

### Physicochemical Determinants

Many previous analyses of GN activity have investigated the physicochemical characteristics of compounds necessary to enable the crossing of the GN outer membrane, often focusing on their hydrophobicity (log *P*) and MW and the rigidity of the structures.^14−16,18^ We therefore re-examined all of the chemical transformations that enhance GN permeation for systematic changes in these parameters. The rigidity of a given molecule was assessed by determining its number of rotatable bonds.

Table 6 shows that, while trends are observed for transformations that lead to the addition or removal of a specific group, trends between different types of transformations are usually dissimilar. For example, all transformations in which a primary amine is added reduce the hydrophobicity, whereas the addition of aryl chloride increases hydrophobicity. On average, only weak trends are seen overall, in which the log *P* is slightly raised along with MW, while molecules with improved GN activity are slightly more rigid.

**Table 6:** Changes in Molecular Weight (MW), Hydrophobicity (log *P*), and Molecular Flexibility (Number of Rotatable Bonds) Associated with the Molecular Substitutions We Identified to Enhance GN activity or Permeation for ChEMBL Datasets 1 (pMIC) and 2 (Permeation)

			dataset 1				dataset 2			
main moiety	+ /–	opposite moiety	pMIC repeats	ΔMW	Δlog *P*	Δno. rotatable bonds	permeation repeats	ΔMW	Δlog *P*	Δno. rotatable bonds
primary amine	addition	ether	225	–17.82	–0.13	–0.69	179	–18.88	–0.1	–0.7
primary amine	addition	carbonyl	655	–13.16	–0.13	–0.3	396	–17.54	–0.01	–0.35
primary amine	addition	secondary amine	2267	–13.61	–0.34	–0.94	2049	–14.29	–0.37	–1
lactone	removal	secondary amine	5	39.27	–0.16	0.6	3	44.74	0.19	0.33
lactone	removal	amine, tertiary	71	39.36	0.46	0.85	39	46.04	0.78	0.69
ester (carboxylate ester)	removal	secondary amine	21	4.37	0.28	–0.19	13	1.8	0.08	–0.31
ester (carboxylate ester)	removal	carboxamide	28	–21.43	–1.51	–2.21	26	–23.58	–1.59	–2.31
ester (carboxylate ester)	removal	ether	26	–0.97	0.39	0.27	13	1.25	0.13	0.38
ester (carboxylate ester)	removal	primary amine	36	–20.52	–0.19	–0.64	25	–17.18	–0.08	–0.76
ester (carboxylate ester)	removal	tertiary amine	106	37.1	0.48	0.42	51	31.21	0.49	0.25
carbonyl	removal	aryl chloride	48	0.96	0.58	–0.94	16	13.08	0.94	–0.88
nitrile	removal	ether	77	11.85	–0.13	1.01	34	13.28	–0.48	0.94
carboxamide	removal	thiophene	8	9.1	2.35	–1	1	75.92	2.33	–1
carboxamide	removal	carboxylic acid	42	–4.82	0.44	0.71	1	–0.00	1.48	0.00
carboxamide	removal	aryl chloride	5	–15	0.88	–1.2	2	–3.58	1.15	–1.5
thiophene	addition	aniline	16	–15.92	0.77	–0.75	6	–15.65	0.86	–0.67
thiophene	addition	carbonyl	39	40.18	1.34	–0.26	15	47.68	1.39	–0.4
tertiary carboxamide	removal	thiophene	5	11.13	2.11	–0.8	1	75.92	2.33	–1
tertiary carboxamide	removal	secondary carboxamide	35	–22.13	–0.62	0.83	13	–23.51	–0.57	0.54
aryl chloride	addition	nitrile	53	17.6	0.89	0	9	24.96	0.94	0
aryl chloride	addition	tertiary amine	52	2.07	1.15	–0.77	4	18.13	0.81	–1.25
aryl chloride	addition	aryl fluoride	291	16.1	0.55	0	49	14.46	0.55	0
secondary amine	addition	tertiary amine	1066	–15.1	–0.3	–0.05	755	–14.17	–0.33	0.07
secondary amine	addition	ether	203	–13.83	0.21	–0.28	150	–11.58	0.2	–0.3
secondary amine	addition	nitrile	62	4.03	–0.07	0.35	35	12.96	0.02	0.91
tertiary amine	removal	aryl chloride	52	2.07	1.15	–0.77	4	18.13	0.81	–1.25
tertiary amine	removal	alkanol	236	–21.80	–0.44	–0.22	99	–27.61	–0.69	–0.62
α,β-unsaturated carbonyl	removal	thiophene	11	50.58	1.07	0.45	6	56.97	1.33	0
α,β-unsaturated carbonyl	removal	secondary amine	25	–9.1	0.05	0.96	2	43.07	0.21	0
α,β-unsaturated carbonyl	removal	aryl chloride	7	28.52	0.75	0	2	30.48	0.7	0
aryl fluoride	addition	nitrile	64	–6.57	0.2	–0.03	16	–6.59	0	–0.06
aryl fluoride	addition	tertiary amine	56	–10.79	0.49	–0.91	17	–19.07	0.04	–1
aryl fluoride	addition	alkanol	81	7.36	0.74	–0.35	23	3.11	0.29	–0.61
*average*				*3.67*	*0.43*	*–0.23*		*11.3*	*0.45*	*–0.37*

Taken together, these findings confirm that simple physicochemical parameters are not well suited to differentiate between GN-active or permeable and non-permeable drugs due to their low degree of separation. According to our results, the presence or absence of specific chemical moieties, by contrast, serves as a much better predictor of GN activity and enables an interpretation of GN activity or permeability on the basis of chemical properties. This is in agreement with recent meta-studies of GN compound uptake, where no consensus about the ideal physicochemical features optimizing permeability has been reached.^19^

## Conclusion

The development of new broad-spectrum antibiotics with sufficient activity against both Gram-positive and Gram-negative pathogenic bacteria is essential to address the drug-resistance problem emerging across a broad range of bacterial infections. Drug permeation across the Gram-negative cell envelope has been recognized as the primary obstacle in achieving a sufficient drug concentration and target activity in Gram-negative bacterial pathogens and is a result of a complex interplay of multiple factors, including outer-membrane translocation and efflux.^4−6,10^ Although previous attempts to derive simple rules determining activity or permeation have had some success, there is, so far, no consensus among these studies regarding the roles of molecular features, which is likely primarily due to limitations in the amount of permeation data analyzed.^12,14−18^

In the absence of large intracellular drug concentration datasets, we set out to make use of sizable publicly available bacterial MIC datasets, rigorously curated to reduce noise from different experimental procedures and to optimally represent the effect of GN bacterial permeation. ML was used to expand the available dataset by synthetically generating new compound–probability pairs from the known inhibition data. This dataset, containing 2.6M compounds in total, was then analyzed for chemical features that influence GN activity by using matched molecular pair analysis. The results were validated by analyzing available *in vitroE. coli* and *S. aureus* inhibition data from ChEMBL.

Our analysis highlights a number of molecular substructures that are consistently associated with enhanced GN activity. These moieties include various amines, thiophenes, and halides, and thus potentially expand the medicinal chemistry toolbox beyond the previously suggested addition of terminal amine groups to enhance GN permeation.^18^ We found that 86% of our predicted molecular modifications indeed improve *E. coli* growth inhibition in the independently analyzed MIC data from ChEMBL. Furthermore, in 76% of the cases they promote GN bacterial permeation, according to our curated permeation proxy.

In 2017, Richter et al. showed that ionizable nitrogen, or more specifically, primary amines exert a positive effect on GN permeation by using cellular concentration data obtained through LC-MS/MS measurements.^18,20^ They also found that molecular globularity and the number of rotatable bonds are negatively correlated to permeation; i.e., flat, rigid compounds displayed improved GN uptake. Our analysis highlights a wide range of amine functions that improve GN activity, which indicates that our computational model can successfully predict GN permeability and suggest experimentally validated molecular modifications to enhance uptake. Overall, we also find a tendency of the more GN-active molecules to possess fewer rotatable bonds, i.e. a greater rigidity; however, the effect is moderate. A slightly positive correlation between the molecular hydrophobicity and GN activity is observed in our study, which is in keeping with two previous LC-MS/MS studies that directly determined cellular accumulation.^30,31^ Our approach therefore confirms several previous findings from experiments on compound permeation, but at the same time substantially widens the range of available modifications that can be made to a drug candidate to enhance its activity in GN bacteria.

The 2705 individual structural transforms that improve GN activity (Table S1C) provide specific examples of compounds and modifications that are optimizing a given core structure for GN uptake. In order to aim for broader applicability, we analyzed those transforms more deeply, in terms of recurring functional groups and moieties, to identify moiety exchange relationships (Table 4). These generalized moiety exchanges may serve as a resource for medicinal chemists to guide optimization and synthesis of other core molecules in a more general way.

Notable current limitations of our work are the derivation of our methodology from data on two exemplar bacterial species, *S. aureus* and *E. coli*, to maintain minimal noise levels. It is therefore not yet possible to predict how well these results generalize across different GN species. Furthermore, the compounds have not yet been grouped into their likely mode of action or bacterial target. This could inform, for example, if permeation across the cytoplasmic membrane is necessary, which may influence the optimal chemistry needed for permeation to the target. An additional point our analysis cannot fully address at the moment is the role of other factors that may lead to increased GN activity, beyond structural or physicochemical changes between the compounds that influence drug uptake, such as differences in the chemical stability of the transformed compounds. These points will be addressed in future studies, depending on the availability of publicly accessible datasets of sufficient size and quality.

## Experimental Section

The overall workflow of the approach we followed is summarized in Figure 3. Publicly available minimal inhibitory concentration (MIC) data was obtained and carefully curated to express it as data suitable to serve as proxy for GN permeation (see below).^29,32−34^

An initial cycle of MMPA showed that the curated initial dataset was not large enough to comprise a sufficient number of significant molecular structural changes (transforms) that would allow us to interpret the GN activity or permeation data using MMPA alone. We therefore used ML to generate a large amount of additional synthetic data based on the curated dataset, in an approach similar to that described by Fu et al.^35^ We trained a ML model on the curated compounds and predicted a GN-activity score, reflecting a proxy for the probability of GN permeation, for 2.6M new molecules. We then performed MMPA on 60 000 rationally selected molecules from the expanded dataset to identify transforms that have a substantial influence on GN activity and emerge from a statistically significant number of structural modifications to molecular cores. Further analysis of the differing molecular structures in these transforms yielded the major chemical determinants of GN activity. Following independent validation of the resulting transforms by comparing them to data retrieved from the ChEMBL database, our findings suggest that some of the key determinants identified in our study can serve as rules to guide molecular design for enhanced GN activity and permeation.^29^

### Data Curation

MIC data for compounds acting on GN and GP pathogens was retrieved from the Collaborative Drug Discovery (CDD) public database, which contains data on antibiotic activity from a variety of public and proprietary sources, as well as from the Community for Open Antimicrobial Drug Discovery (CO-ADD), an open access database which hosts screening results for compounds with potential antimicrobial activity.^29,32−34^ To reduce noise levels in data arising from different types of measurements and on different species, we selected two paradigmatic pathogens as representatives for each type of cell envelope: *E. coli* for GN bacteria and *S. aureus* for GP bacteria. By focusing on these two species, we obtained the largest datasets for individual species, which at the same time originate from only a small range of different experimental procedures.

Importantly, the molecular targets for existing antibiotics inside the cells of *S. aureus* and *E. coli* are commonly thought to be homologous. This assumption is underpinned by the action of known broad-spectrum antibiotics that act on analogous targets across both GN and GP bacteria such as β-lactams, quinolones, tetracyclins, and other antibiotic classes.^36−38^ In turn, this means that reduced activity levels of individual antibiotics within GN bacteria are likely to be caused by their diminished ability to permeate the GN cell envelope, and in this way the MIC data can be transformed to yield a proxy for permeability.

Accordingly, we curated the retrieved MIC data (Table 2, initial data) to optimally represent GN permeability. Compounds were split into two groups: those that are active in GN bacteria and can therefore permeate the GN cell wall and those that cannot. An activity threshold was imposed at pMIC ≤ 5 (pMIC = −log_10_(MIC [μM] × 10^–6^)). This cutoff corresponds to an MIC value of 10 μM, which represents a lower threshold of high to medium activity for small-molecule inhibitors, removing compounds which display only low activity. A similar boundary has been used in previous quantitative structure–activity relationship (QSAR) studies, such as that by Tripathy et al.^39^ Furthermore, imposing an activity threshold allowed us to convert the continuous data contained in MIC or pMIC values into binary labels for every compound. This enabled the use of a classification machine learning model which entails a range of advantages, including computational performance and prediction accuracy. By contrast, regression models often result in a higher degree of data overfitting and therefore reduce the quality of predictions.

Using this threshold, labels were assigned to all compounds: “1”, the compound permeates both GN and GP cell walls and is active against both types of bacteria; “0”, the compound permeates the GP but not the GN cell wall (or is otherwise not active against GN bacteria) and shows activity only on GP bacteria.

By selecting compounds that are proven to be active against *S. aureus*, but not necessarily against *E. coli*, a differentiating property was created, in which the difference between compounds labeled as “0” and “1” and the barrier to activity against *E. coli* are most likely to be caused by different permeation rates across the cell envelope (including both low inward uptake and active efflux).

Table 1 summarizes the compound curation and labeling procedure. Based on the employed criteria, only compounds that show at least medium-level activity against *S. aureus*, and which have also been tested against *E. coli* (as either actives or non-actives), are retained and labeled.

### Matched Molecular Pair Analysis

MMPA compares the properties of pairs of molecules that differ only by a small structural change, known as the transformation (Figure 2).^40^ MMPA can be applied to large molecular datasets, generating a high number of pairs, and is able to compare multiple molecular properties at once, which makes MMPA a convenient multi-parameter optimization tool. Conventionally, MMPA is used to analyze compounds with associated experimentally measured property or activity values.

Similar approaches have previously been used in a study of the prediction of transform activity in an absorption, distribution, metabolism, and excretion (ADME) dataset, where a QSAR model was used as a scoring function for MMPA.^41^ Additionally, a recent study predicted log *D*_7.4_ values by combining ML and MMPA on a dataset expanded by synthetically generated data, validating this type of approach.^35^ By linking changes in structure to changes in property, MMPA has been shown to be able to act as an inverse QSAR technique in a way that allows chemically intuitive deconvolution of structure–activity relationships to be performed easily.^42^ MMPA was performed in MCpairs (Medchemica Limited, Macclesfield, UK, 2020) and employed both maximum common substructure and fragment and index methods, with settings described previously by Lukac et al.^43^

### Synthetic Data Generation

The validity of ML models and analysis methods such as MMPA relies on the availability of large and diverse datasets. Publicly available bacterial permeation datasets are often too small to be leveraged in a reliable way. In comparison, the permeation-proxy dataset we collected, curated from inhibition activity, is to our best knowledge currently the largest of its type and allowed us to build a reliable ML predictor of GN activity. Alongside its use as a predictor for the activity of any given new molecule, the model also allows for the synthetic expansion of the initial dataset by predicting the learned property on large collections of new compounds. Importantly, it allows the underlying chemical features to be amplified and detected by further statistical analysis, even in the case where the original datasets are of limited size.

In the pioneering work by Stokes et al.,^22^ a ML method, *Chemprop*, was recently used to discover structurally new antibiotics, including a GN-active compound named Halicin. The *Chemprop* model uses a directed-message-passing neural network to aggregate information from features of local atoms and bonds for every molecule in the training set, represented as a graph. In our case, this molecular information was combined with the associated activity/permeation label for each compound which was derived from the input datasets. The *Chemprop* model, after training, is then capable of predicting the learned property, i.e., activity, in new molecules that are not part of the initial training and test sets.^22,44^

To generate synthetic data representing a proxy for GN permeability, we used the labeled and curated compounds as classification data, setting aside 15% of the compounds with balanced class distribution as a test set. Training and testing was performed using *Chemprop*.^22,44^ Before training our model, a built-in method for hyperparameter optimization, which uses a Bayesian optimization algorithm, was used on the training data. To optimize the prediction, we trained an ensemble of five ML models and carried out 5-fold cross-validation. To promote wider generalization of the ensemble, the molecular representations were supplemented by additional physicochemical descriptors as calculated by the chemoinformatic software package *rdkit*.^45^ Both hyperparameter optimization and training were carried out on a local GPU cluster.

The trained model was then applied on independent external datasets to predict the GN activity of a given compound. We used three external datasets: ENM_1, ENM_2, and ENM_3, originating from the chemical synthesis company Enamine, consisting of about 2.6 million compounds altogether. The datasets ENM_1, ENM_2, and ENM_3 correspond to “HTS”, “Advanced”, and “Premium”, respectively, in the Enamine documentation.^46^ These datasets represent a wide range of physicochemical properties as well as a large variety of functional groups, covering well the chemical space of the initial compounds (Figures S1 and S2). The predicted GN-activity score was then used as the synthetically generated activity measure in the subsequent MMPA of these new datasets.

Due to the high computational cost of identifying molecular pairs in the large dataset by MMPA, we pre-processed the resulting synthetic data as follows. The similarity of the compounds was calculated by converting all compounds into extended connectivity fingerprints, where molecular structures are represented by bits in a binary vector.^47^ The structures were then compared to each other by using the Jaccard–Tanimoto coefficient, which computes intersections of bits in the two binary vectors.^48^ To optimize the selection of molecular pairs, i.e., compounds with a common core and therefore high molecular similarity, compounds within each dataset that exhibited no or low molecular similarity (at a 50% threshold) to the 10 000 highest-scoring compounds for permeability were discarded. From the remaining compounds (with above 50% similarity to the top 10 000 compounds), the 10 000 molecules with the lowest GN-activity score were retained alongside the 10 000 highest-scored compounds. In this way, we maximized both the number of molecular pairs among the compounds in the pre-processed set and their score difference. This selection resulted in 20 000 compounds for each of the three ENM datasets, totaling 60 000 compounds.

## Supporting Information Available

Trained *Chemprop* model (ZIP)
Molecules contained in the initial dataset (SMILES) and assigned labels (CSV)
Supporting Figures S1, S2, and S3, showing synthetic data generation and distributions of pMIC caused by transforms from Table 5 (PDF)
Supporting Table S1A–C (XLSX)

## Abbreviations Used

CDD: Collaborative Drug Discovery
CO-ADD: Community for Open Antimicrobial Drug Discovery
GN: Gram-negative
GP: Gram-positive
LC-MS/MS: liquid chromatography–tandem mass spectrometry
MIC: minimum inhibitory concentration
ML: machine learning
MMPA: matched-molecular-pair analysis
QSAR: quantitative structure–activity relationships
WHO: World Health Organization
