# Supplementary material for: Data-Driven Derivation of Molecular Substructures That Enhance Drug Activity in Gram-Negative Bacteria
Source: J Med Chem. 2022 Apr 15;65(8):6088–99. doi: 10.1021/acs.jmedchem.1c01984 (PMC9059115; doi:10.1021/acs.jmedchem.1c01984)
Supplement: Supplementary file 3 — jm1c01984_si_003.pdf [file jm1c01984_si_003.pdf]

Supporting Information

Data-driven derivation of molecular substructures that enhance drug activity in Gram-negative bacteria

Dominik Gurvic[a], Andrew G. Leach[b, c], and Ulrich Zachariae[a,*]

[a] Computational Biology, School of Life Sciences, University of Dundee, Dow Street, Dundee, DD1 5EH, United Kingdom.
[b] Division of Pharmacy and Optometry, University of Manchester, Oxford Road, Manchester, M13 9PL, United Kingdom.
[c] Medchemica Limited, Mereside, Alderley Park, Macclesfield, SK10 4TG, United Kingdom.
*Corresponding author u.zachariae@dundee.ac.uk

Table of Contents

Synthetic Data Generation 2
Distributions of pMIC caused by transforms from Tab. 5 3

Synthetic Data Generation

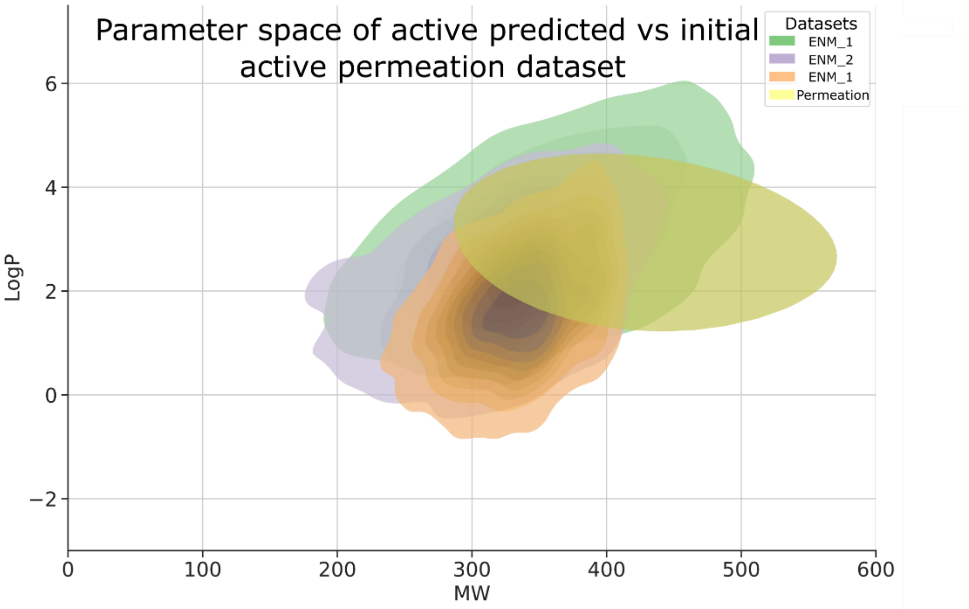

Fig. S1: Chemical space, represented by LogP and MW parameters, for three datasets: ENM_1--ENM_3 used to predict permeation compared to the initial permeation dataset (GN-actives only).

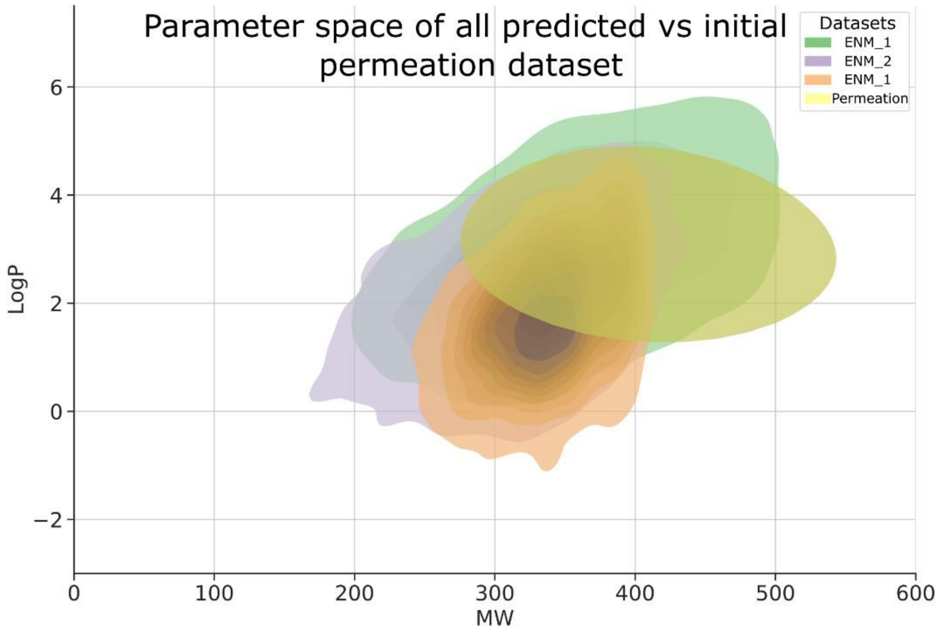

Fig. S2: Chemical space, represented by LogP and MW parameters, for three datasets: ENM_1--ENM_3 used to predict permeation compared to the initial permeation dataset (all data).

Distributions of pMIC caused by transforms from Tab. 5

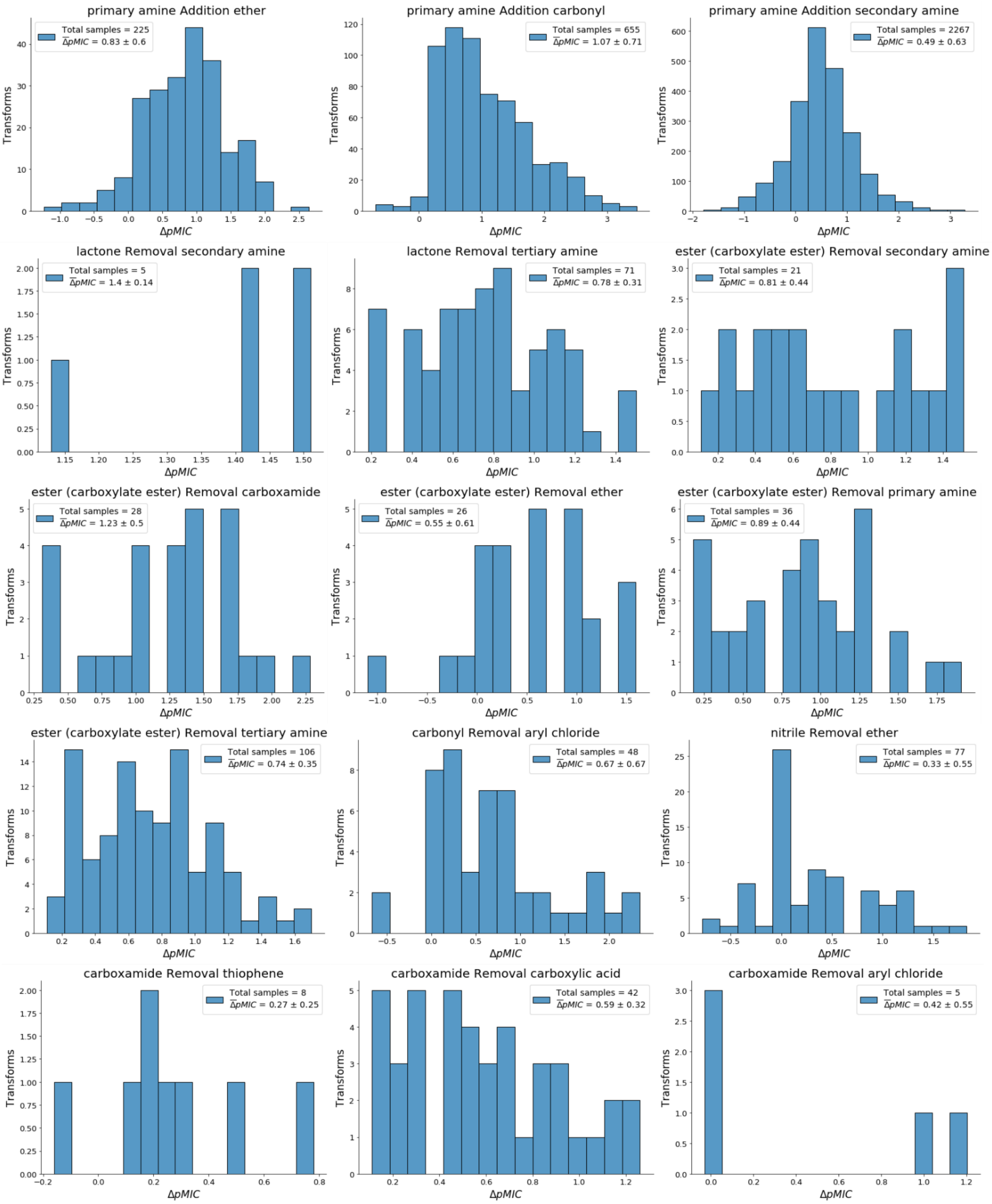

Fig. S3: Distributions of change in pMIC caused by every transform from Tab. 5. Histogram titles correspond to first three columns of Tab. 5: 'Main Moiety', '±', 'Exchange Moiety' for every transform. (part 1)

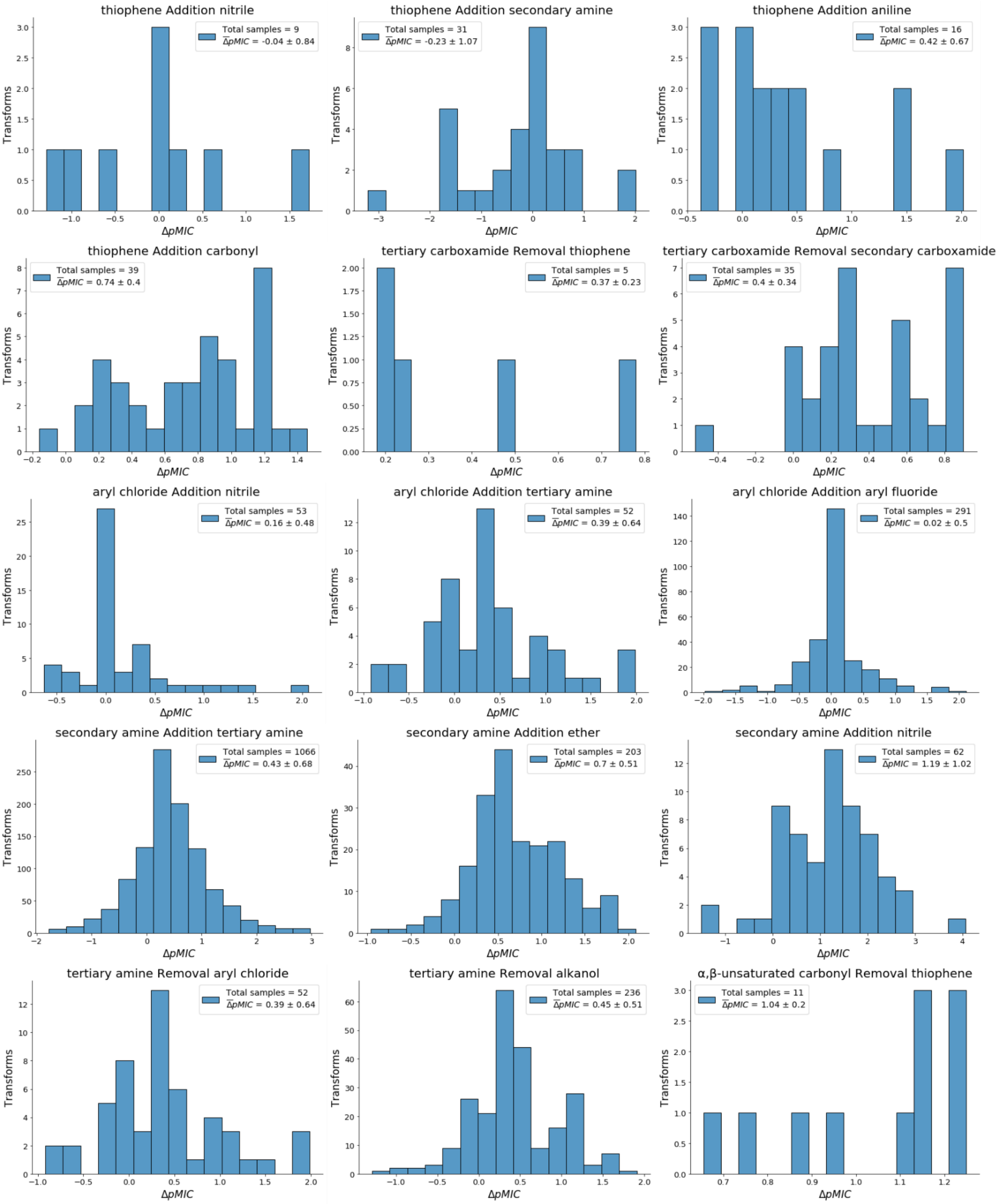

Fig. S3: Distributions of change in pMIC caused by every transform from Tab. 5. Histogram titles correspond to first three columns of Tab. 5 : 'Main Moiety', '±', 'Exchange Moiety' for every transform. (part 2)

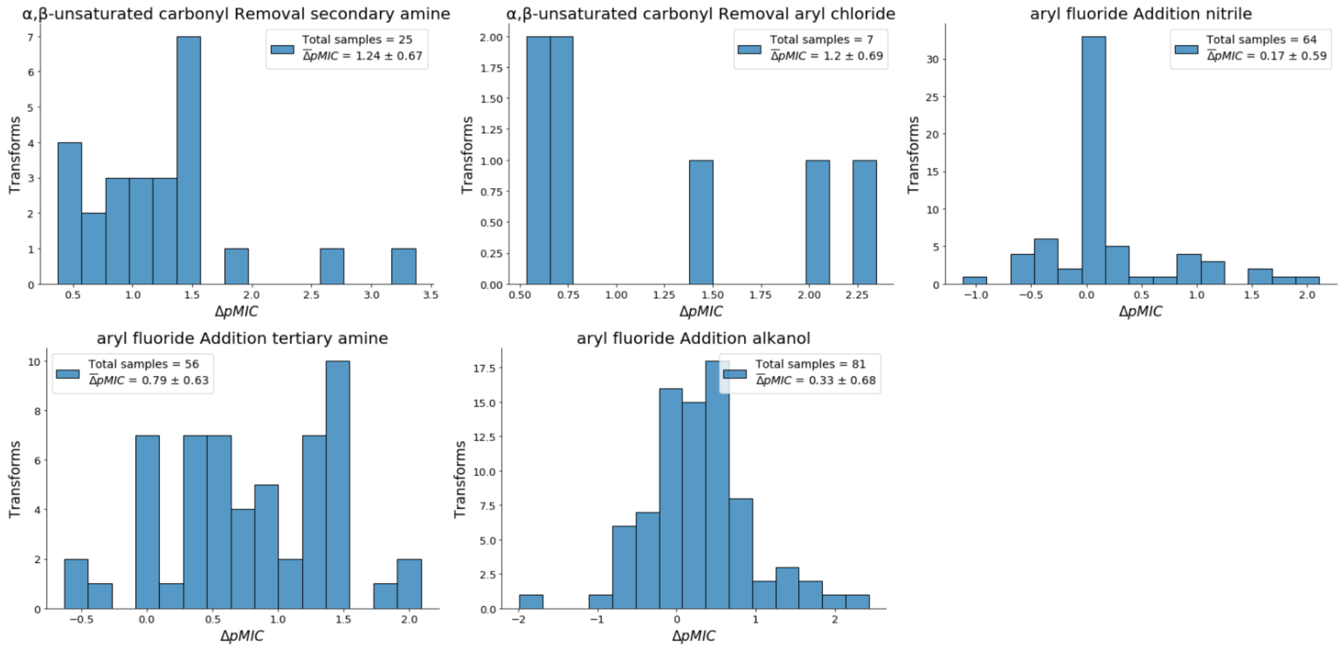

Fig. S3: Distributions of change in pMIC caused by every transform from Tab. 5. Histogram titles correspond to first three columns of Tab. 5 : 'Main Moiety', '±', 'Exchange Moiety' for every transform. (part 3)
